# Post-Transcriptional Regulation of Iron Homeostasis in *Saccharomyces cerevisiae*

**DOI:** 10.3390/ijms140815785

**Published:** 2013-07-30

**Authors:** María Teresa Martínez-Pastor, Rosa de Llanos, Antonia María Romero, Sergi Puig

**Affiliations:** 1 Departamento de Bioquímica y Biología Molecular, Universitat de València, Ave. Doctor Moliner 50, Burjassot E-46100, Valencia, Spain; E-Mail: maria.teresa.martinez@uv.es; 2 Departamento de Biotecnología, Instituto de Agroquímica y Tecnología de Alimentos (IATA), Consejo Superior de Investigaciones Científicas (CSIC), Ave. Agustín Escardino 7, Paterna E-46980, Valencia, Spain; E-Mails: rodella@iata.csic.es (R.L.); am.romero@iata.csic.es (A.M.R.)

**Keywords:** Iron, yeast, post-transcriptional regulation, mRNA decay, alternative 3′ end processing, Cth1, Cth2, Rnt1

## Abstract

Iron is an essential micronutrient for all eukaryotic organisms because it participates as a redox cofactor in a wide variety of biological processes. Recent studies in *Saccharomyces cerevisiae* have shown that in response to iron deficiency, an RNA-binding protein denoted Cth2 coordinates a global metabolic rearrangement that aims to optimize iron utilization. The Cth2 protein contains two Cx_8_Cx_5_Cx_3_H tandem zinc fingers (TZFs) that specifically bind to adenosine/uridine-rich elements within the 3′ untranslated region of many mRNAs to promote their degradation. The Cth2 protein shuttles between the nucleus and the cytoplasm. Once inside the nucleus, Cth2 binds target mRNAs and stimulates alternative 3′ end processing. A Cth2/mRNA-containing complex is required for export to the cytoplasm, where the mRNA is degraded by the 5′ to 3′ degradation pathway. This post-transcriptional regulatory mechanism limits iron utilization in nonessential pathways and activates essential iron-dependent enzymes such as ribonucleotide reductase, which is required for DNA synthesis and repair. Recent findings indicate that the TZF-containing tristetraprolin protein also functions in modulating human iron homeostasis. Elevated iron concentrations can also be detrimental for cells. The Rnt1 RNase III exonuclease protects cells from excess iron by promoting the degradation of a subset of the Fe acquisition system when iron levels rise.

## 1. Introduction

Iron (Fe) is an indispensable element for all eukaryotes and the vast majority of prokaryotes because it serves as a redox active cofactor in energy generation (mitochondrial respiration, photosynthesis) and in the biosynthesis of major cell components: proteins (ribosomes, several amino acids), nucleic acids (deoxyribonucleotides, DNA) and lipids (unsaturated fatty acids, sterol and sphingolipids). Despite Fe being abundant, its extremely low solubility at a physiological pH compromises its bioavailability. In fact, Fe deficiency anemia is the most extended and common nutritional disorder in the world [1,2]. The same redox properties that enable Fe to function as an essential cofactor in many metabolic pathways can also be detrimental for cells. When present at elevated concentrations, Fe can participate in Fenton-type reactions that promote the formation of powerful reactive oxygen species, including hydroxyl radicals that damage cells at the levels of lipid peroxidation, protein oxidation and carbonylation, and DNA mutagenesis and destabilization [3]. Recent studies have revealed that Fe excess can be toxic for yeast cells, even in the absence of oxygen, probably due to the activation of the sphingolipid signaling and synthesis pathway [4,5]. Consequently, alterations in Fe homeostasis bring about multiple human diseases, including hereditary hemochromatosis, Friedreich’s ataxia and aceruloplasminemia (reviewed in [6–9]). Hence, living organisms have developed sophisticated regulatory mechanisms that modulate the expression of the genes involved in Fe sensing, acquisition, distribution, storage, recycling and utilization to achieve an appropriate Fe balance.

In mammals, cellular Fe homeostasis is mostly controlled through post-transcriptional mechanisms. Two Fe-regulatory proteins, IRP1 and IRP2, control the expression of the genes involved in cellular Fe homeostasis by specifically interacting with stem-loop mRNA structures, termed Fe-responsive elements or IREs (reviewed in [10,11]). The recognition and binding of IRPs to IREs depends on the cellular Fe status. When Fe levels are low, both IRPs bind to IREs, whereas no interaction is observed when Fe is in excess. The position of the IRE on the transcript is crucial to determine the fate of IRP-bound mRNA. The transcripts encoding the proteins required for Fe uptake, such as transferrin receptor TfR1 and Fe transporter DMT1, contain IREs at the 3′ untranslated region (UTR). When Fe is scarce, the IRP/IRE complex assembled at the 3′ UTR prevents mRNA degradation, leading to an increase in the TfR1 and DMT1 protein levels that stimulates cellular Fe acquisition. Furthermore, the mRNAs encoding proteins that participate in Fe storage (ferritin H and L), Fe export (ferroportin) or Fe utilization (erythroid aminolevulinate synthase, mitochondrial aconitase) display an IRE at the 5′ UTR. In response to Fe deficiency, the IRP/IRE complexes assembled at the 5′ UTR of these transcripts inhibit translation. When cellular Fe concentration increases, IRPs are released from the 5′ IRE and protein synthesis proceeds, leading to the accumulation of ferritin and ferroportin, which protect cells from toxic Fe levels. Importantly, enterocytes accumulate ferroportin in response to Fe deficiency due to the expression of a ferroportin transcript that lacks the 5′ IRE [12]. Recent global studies have extended potential IRP-regulated genes even beyond Fe homeostasis [13]. The mechanisms modulating the activity of both IRPs in response to fluctuations in cellular Fe availability differ. Under Fe-sufficient conditions, IRP1 assembles an Fe-S cluster that confers IRP1 aconitase activity. Upon Fe scarcity, IRP1 loses its Fe center and its aconitase activity, but undergoes extensive conformational changes that enable apo-IRP1 to interact with IREs [14]. The IRP2 protein does not bind Fe, but its stability is regulated by Fe abundance. When cellular Fe levels rise, an E3 ubiquitin ligase complex containing the FBXL5 protein promotes IRP2 ubiquitination and proteasomal degradation. The FBXL5 protein contains a hemerythrin domain that stabilizes the protein upon Fe-binding. When intracellular Fe decreases, the FBXL5 protein is degraded, leading to IRP2 accumulation [15,16].

The budding yeast *Saccharomyces cerevisiae* is an excellent model organism that has contributed tremendously to elucidate the molecular mechanisms that eukaryotic cells utilize to respond to fluctuations in Fe bioavailability. As shown for mammalian IRP1, yeast cells perceive Fe deficiency and Fe excess by alterations in the Fe-S cluster synthesis rate [17,18]. When Fe-S biosynthesis proceeds, the Fe-regulated Aft1 (and probably Aft2) transcription factor perceives a mitochondrial Fe signal through a Grx3/Grx4-dependent mechanism that diminishes the transcription of Fe starvation response genes [19–21]. In response to Fe scarcity, Fe-S synthesis decreases and Aft1 (and probably Aft2) accumulates in the nucleus, binds to specific Fe-response elements termed FeRE, and activates the transcription of around 25 genes that function in Fe homeostasis and are denoted the Fe regulon (reviewed in [22,23]). Among other processes, the coordinated action of Aft1 and Aft2 stimulates Fe uptake by: inducing the expression of a family of metalloreductases (*FRE1-5*), a high-affinity Fe uptake complex (*FET3/FTR1*), the cell wall mannoproteins involved in Fe acquisition (*FIT1-3*), and a family of Fe siderophore transporters (ARN1-4); activating Fe mobilization from vacuolar stores by promoting the transcription of a vacuolar metalloreductase (*FRE6*) and two Fe transport systems (*FET5/FTH1; SMF3*); initiating a profound metabolic remodeling of Fe-dependent processes by increasing the expression of two RNA-binding proteins denoted Cth1 and Cth2 (reviewed in [24–26]). Under Fe replete or high Fe conditions, yeast cells increase the rate of mitochondrial Fe-S cluster biosynthesis, which sends out an Fe signal that is perceived and activates the nuclear Yap5 transcription factor [18]. Upon sensing the Fe signal, Yap5 activates, among other targets, the transcription of *CCC1*, a vacuolar Fe importer that protects cells from Fe toxicity by triggering Fe storage [27,28].

## 2. Post-Transcriptional Regulation of Gene Expression in Response to Iron Deficiency

### 2.1. Cth1 and Cth2 RNA-Binding Proteins Post-Transcriptionally Regulate Iron-Dependent Processes

Among the genes transcriptionally activated by Aft1 or Aft2 in response to Fe deficiency, *CTH2* (also known as *TIS11*) is one of the few members of the Fe regulon encoding a soluble, non membrane-associated protein [29–32]. The most remarkable feature of the Cth2 protein is the presence of two tandem zinc fingers (TZFs) of the Cx_8_Cx_5_Cx_3_H type (x being a variable amino acid) separated by 18 amino acids. Experiments using the yeast three-hybrid assay, a method that detects RNA-protein interactions *in vivo* [33], have demonstrated that Cth2 binds in a TZF-dependent manner to the adenosine/uridine-rich elements (AU-rich elements or AREs) present in the 3′ untranslated region (UTR) of multiple mRNAs, including *SDH4* (a subunit of succinate dehydrogenase), *ACO1* (aconitase), and *WTM1* (ribonucleotide reductase nuclear anchor) [31,34]. Transcription shut-off experiments have demonstrated that upon binding Cth2 promotes the destabilization of ARE-containing transcripts as the half-lives of *ACO1* and *SDH4* mRNAs decrease by around 50% under Fe-deficient conditions, but only when cells express a functional Cth2 protein [31]. Interestingly, the 3′ UTR of *SDH4* mRNA confers Cth2-mediated Fe regulation to an Fe-independent transcript [31]. The integrity of both the AREs at the transcript and the TZFs in Cth2 is indispensable for mRNA binding and turnover as the mutations in either the AREs or the TZFs diminish or totally abolish the RNA-protein interaction and transcript degradation [31,34]. All these data suggest that Cth2 is a post-transcriptional regulator of gene expression which promotes the ARE-mediated decay (AMD) of a subset of mRNAs in response to Fe deficiency.

In addition to *CTH2*, the *S. cerevisiae* genome harbors a second gene, denoted *CTH1*, whose product shares an overall 46% identity and 59% similarity to Cth2, as well as 79% identity at the TZFs [31,35]. As shown for Cth2, Cth1 also stimulates the AMD of multiple Fe-related mRNAs [36]. One important difference between the *CTH1* and *CTH2* genes lies in their distinct expression pattern. *CTH2* mRNA is not detected, or is extremely lowly expressed, under normal conditions (meaning the cells exponentially growing under 2% glucose and Fe-sufficient conditions), whereas in these circumstances, the *CTH1* gene displays basal levels of expression [30,31,36,37]. Upon Fe limitation, transcription factors Aft1 and Aft2 cooperate to bind to FeREs within the promoter of both the *CTH1* and *CTH2* genes and activate their transcription [31,34]. Despite this, the Cth2 mRNA and protein levels sharply rise during the progress of Fe deficiency, whereas Cth1 mRNA and protein abundance remain low [31,36]. The relative relevance of the *CTH1* and *CTH2* genes in the response of yeast cells to Fe deprivation is evidenced by the growth phenotypes observed for the corresponding deleted strains. Those cells lacking *CTH2* display a major growth defect under Fe-deficient conditions, whereas the deletion of *CTH1* by itself does not lead to any growth defect under low Fe conditions, but exacerbates the phenotype displayed by the *cth2Δ* mutant, strongly suggesting that both proteins contribute to the cellular adaptation to Fe limitation [31].

Genome-wide transcriptome experiments using DNA microarrays have shown that under Fe-deficient conditions, Cth2 is responsible for the down-regulation of more that 200 mRNAs, whereas Cth1 contributes to the decrease approximately 60 transcripts [31,36]. Around 40% of these down-regulated genes contain consensus AREs in their 3′ UTRs and can be considered direct Cth1/Cth2 targets, whereas the remaining decreased transcripts may be due to indirect effects of the Cth1/Cth2 function or the Cth1/Cth2-binding to noncanonical AREs. Importantly, the majority of ARE-containing transcripts regulated by Cth1/Cth2 during Fe deficiency encode the proteins involved in metabolic pathways which directly or indirectly depend on Fe as a cofactor. Since mitochondrial respiration consumes large amounts of Fe, Cth1 and Cth2 primarily target the degradation of numerous transcripts whose products participate in the mitochondrial electron transport chain and the tricarboxilic acid cycle, such as *SDH4* and *ACO1*. Thus, one of the most significant alterations that both Cth1 and Cth2 seem to perform upon Fe depletion is a shift to fermentative metabolism. In addition to respiration, Cth2 down-regulates the mRNAs encoding those proteins that participate in multiple Fe-requiring pathways, such as the biosynthesis of some amino acids (leucine, lysine, methionine, glutamate), lipids (unsaturated fatty acids, ergosterol, sphingolipids), cofactors (lipoic acid), heme and some 4Fe-4S containing enzymes [31,36]. Under low Fe conditions, Cth2 also inhibits Fe storage by promoting the destabilization of *CCC1* mRNA, whose product imports Fe into the vacuole [28,31]. Recent data have demonstrated that, in addition to down-regulating nonessential Fe-consuming pathways, under Fe scarcity, the Cth1 and Cth2 proteins stimulate the activity of essential Fe-dependent enzymes, such as ribonucleotide reductase (RNR) [34]. RNR is an essential enzyme required for DNA synthesis that catalyzes the conversion of ribonucleotides into the corresponding deoxy forms (reviewed in [38,39]). Upon Fe limitation, Cth1 and Cth2 promote the degradation of *WTM1* mRNA, which encodes a nuclear anchor of the small RNR subunit. The drop in the Wtm1 protein levels mediated by Cth1 and Cth2 facilitates the assembly of an active RNR enzyme in the cytoplasm and promotes deoxyribonucleotide synthesis [34]. Thus, in response to Fe limitation, the Cth1 and Cth2 proteins optimize cellular Fe utilization by decreasing Fe storage and utilization in nonessential Fe-dependent metabolic pathways, and by prioritizing essential Fe-requiring processes, such as DNA synthesis. Besides Cth1/Cth2 post-transcriptional regulation, other transcriptional mechanisms contribute to the metabolic reprogramming that yeast cells undergo during the progress of Fe deficiency. A sharp drop in the metabolites whose synthesis requires Fe, such as α-isopropylmalate and heme, leads to a reduction in the transcription rate of the *LEU1* (Fe-S-containing isopropylmalate isomerase in the branched-chain amino acid biosynthesis) and *CYC1* (heme-containing cytochrome *c*) genes, respectively [40]. Interestingly, the control of the *LEU1* and *CYC1* mRNA levels is conferred by the combination of transcriptional regulation through Fe responsive metabolites and post-transcriptional stability by the Cth1 and Cth2 proteins [40].

### 2.2. Cth2 Is a Nucleocytoplasmic Shuttling Protein

**Figure 1:**
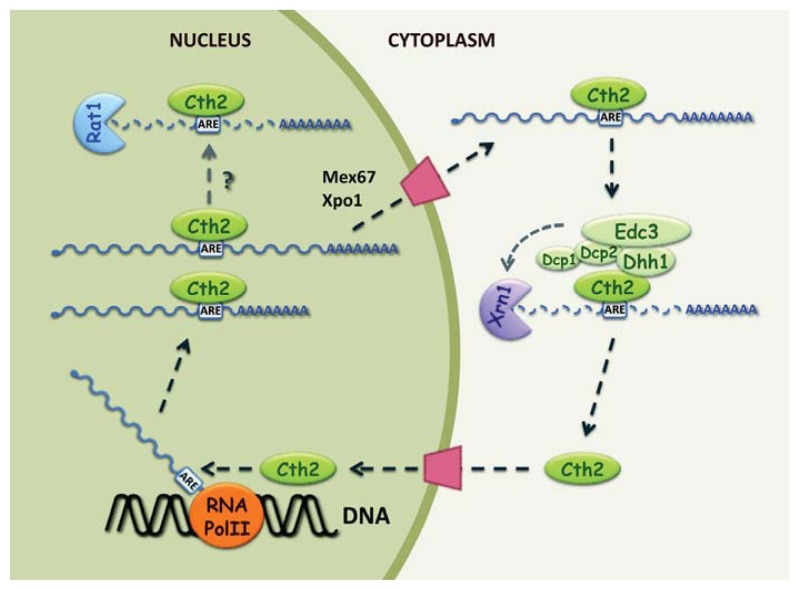
Model for the post-transcriptional regulation of adenosine/uridine-rich element (ARE)-containing transcripts by Cth2 in response to Fe deficiency. This model proposes the following steps for the degradation of the *SDH4* mRNA by Cth2 protein: (1) Docking and recognition: Cth2 co-transcriptionally binds to AREs in the nascent mRNA before polyadenylation occurs; (2) 3′ end processing: Cth2-binding to the AREs partially interferes with normal polyadenylation, leading to the synthesis of extended transcripts, which are preferentially degraded in a Cth2-dependent manner by a 5′ to 3′ exoribonuclease, either Rat1 in the nucleus or Xrn1 after transport to the cytoplasm; (3) Export: Cth2 bound to the transcript is translocated to the cytoplasm via mRNA export pathways that require Mex67 and Xpo1/Crm1; (4) Cytoplasmic degradation: Cth2 interacts with the RNA helicase Dhh1, which recruits the decapping enzymes and Xrn1 to the target mRNA that is degraded from 5′ to 3′; (5) Recycling and nuclear import: After mRNA degradation, Cth2 is released from the decay machinery and can potentially re-enter the nucleus to initiate a new cycle of ARE-mediated decay (AMD).

Localization and biochemical studies using a functional epitope-tagged Cth2 protein have shown that yeast Cth2 localizes to both the nucleus and the cytoplasm of Fe-deficient cells [41,42]. Analyses of truncated versions of the Cth2 fusion protein have failed to identify specific nuclear localization (NLS) and nuclear export signals (NES). Nonetheless, the nuclear import information seems to reside within the TZF domains, which are responsible for mRNA binding [42]. Interestingly, evidence is consistent with a model in which Cth2 can only leave the nucleus when bound to its target transcript [42]. First, Cth2 export to the cytoplasm is impaired in those mutants that are defective in nuclear mRNA export. For instance, the thermosensitive mutants in the essential general mRNA export factor Mex67 accumulate the Cth2 protein in the nucleus at a nonpermissive temperature. Furthermore, Cth2 properly leaves the nucleus in the alleles of the *CRM1*/*XPO1* nuclear export factor, such as *crm1-2* and *crm1-3*, which allow poly(A) RNA export, but do not function in NES-mediated transport. However, temperature-sensitive *xpo1-1* mutants, which are defective in both poly(A) RNA and NES-mediated export, retain Cth2 in the nucleus at a restrictive temperature strongly suggesting that Cth2 translocation to the cytoplasm is solely dependent on RNA export pathways and is independent of NES-mediated transport. Second, the Cth2 proteins mutated in the key cysteine residues of the TZFs, which are unable to bind ARE-containing transcripts, are also retained in the cellular nuclei. Third, inhibition of the mRNA synthesis achieved by the addition of thiolutin also leads to the nuclear accumulation of Cth2. These observations indicate that Cth2 must bind to its target mRNA before leaving the nucleus. Importantly, the addition of exogenous NLS or NES sequences to Cth2, which restrict its localization to either the nucleus or the cytoplasm, results in an impaired Cth2-mediated down-regulation of *SDH4* mRNA and a growth defect under Fe-deficient conditions [42]. This is not due to the tag since inactivated NLS and NES fused to Cth2 properly function in AMD and grow in Fe limitation. Therefore, Cth2 nucleocytoplasmic shuttling is an essential part of the mechanism that the Cth2 (and probably Cth1) protein utilizes to promote AMD in response to Fe deficiency (Figure 1).

### 2.3. Cth2 Promotes mRNA Decay by Alternative 3′ End Processing

Pre-mRNA processing is a universal gene expression step in eukaryotes. Following the transcription by RNA polymerase II in the nucleus, nascent mRNA transcripts undergo a series of co-transcriptional maturation steps before they can be exported to the cytoplasm for translation into proteins. The processing events include the 5′ end capping to add a 7-methylguanosine cap, splicing to remove introns and 3′ end processing, which includes the endonucleolytic cleavage reaction downstream of the coding sequence at the polyadenylation (poly(A)) site, followed by the addition of a nontemplated poly(A) tail. The pre-mRNA 3′ end processing reaction requires the assembly of a large number of protein factors onto specific signal sequences within the 3′ region of the pre-mRNA (reviewed by [43–45]). In *S. cerevisiae*, the poly(A) site is defined by four sequence elements: the AU-rich efficiency element (EE), the A-rich positioning element (PE), the cleavage site, and the upstream (UUE) or downstream (UE) U-rich elements. The AU-rich EE element is located upstream of the cleavage site and it improves the cut. The A-rich PE element location, downstream of the EE and around 10–30 nucleotides upstream of the cleavage site, is critical for efficient 3′ end processing. The cleavage site is generally defined by a pyrimidine followed by multiple adenosines. Finally, UUE and UE are conserved U-rich sequences located near the cleavage site that contribute to the cleavage event. The *S. cerevisiae* 3′ end processing machinery comprises (1) cleavage factor IA (CFIA), a complex including the RNA-binding protein Rna15 and scaffold protein Rna14 that recognizes the A-rich PE to position CPF to cleave the poly(A) site; (2) cleavage factor IB (CFIB) which, in yeast, contains only the RNA-binding protein Nab4 (also known as Hrp1), which interacts with the AU-rich EE site and CFIA to influence the efficiency of the cleavage reaction; (3) the cleavage and poly(A) factor (CPF), including poly(A) polymerase Pap1 which finishes the process after the cleavage reaction. Yeast Pap1 synthesizes a poly(A) tail of 60–80 adenosines in a template-independent manner. After 3′ end processing, the mRNA transcript is targeted to the nuclear pore for mRNA export to the cytoplasm. If any errors in the mRNA transcripts are detected, they are retained in the nucleus and are quickly degraded by quality control mechanisms. The processing of 3′ end pre-mRNA is coordinated with 5′ end capping, transcription, and mRNA stability/translation or export.

The regulatory RNA sequences included in the 3′ UTR can severely influence gene expression by modulating transcript stability, localization, transport or translation efficiency. By using massive RNA sequencing approaches, multiple studies have shown that the majority of *S. cerevisiae* genes express an unprecedented diversity of transcript isoforms which differ in their regulatory elements at their UTRs [46–51]. Many eukaryotic transcripts contain more than one poly(A) signal [52]. Thus, selection of single or alternative poly(A) sites depending on the physiological conditions is emerging as an important mechanism to modulate gene expression [46,47,49,51]. A growing number of proteins have been identified as regulators of the 3′ end transcript processing (reviewed in [43]). In yeast, the Nab4 single component of CFIB modulates *in vivo* the alternative 3′ pre-mRNA processing of multiple mRNAs by specifically binding to AU-rich EEs [53,54]. Both the inactivation and overexpression of Nab4 alter the cleavage site selection of various transcripts, such as those encoding the Ctr2 vacuolar copper transporter, the Sua7 transcription initiation factor TFIIB, Cyc1 or the Gal7 galactose-1-phosphate uridyl transferase [53,55]. Importantly, *nab4* mutants exhibit increased tolerance to copper, which requires the *CTR2* gene, suggesting that alternative Nab4 processing events contribute to yeast cell physiology [53]. Nab4 has also been implicated in mRNA export and nonsense-mediated decay [56,57].

A structure-function analysis of Cth2 has demonstrated that the conserved amino-terminal CR1 region of Cth2, encompassing the first 86 amino acids, is essential for AMD [58]. In addition to normal *SDH4* mRNA levels, the Fe-deficient cells expressing Cth2Δ1-86 mutant protein accumulate 3′ readthrough *SDH4* transcripts [58]. The production of extended transcripts requires Cth2 binding to the target mRNA since they are not present in either the cells expressing Cth2Δ1-86 proteins without functional TZFs or *cth2Δ* mutants. In wild-type cells, the *SDH4* poly(A) sites mostly map to 147–175 nucleotides downstream of the *SDH4* stop codon, which represent normal polyadenylation [41,58]. In contrast, the yeast cells expressing the Cth2Δ1-86 truncated protein display two major poly(A) regions: one similar to that described above and a novel poly(A) distal region located approximately 330 nucleotides downstream of the normal cleavage site [58]. These additional poly(A) sites are consistent with the size estimated for the extended *SDH4* transcripts and they overlap the *CSN9* gene encoding a subunit of the Cop9 signalosome in the antisense direction. It is currently unknown whether *SDH4* transcription through the *CSN9* coding sequence has any effect on the expression of either *SDH4* or *CSN9* genes. An analysis of some of the mutants involved in 3′ end processing revealed that the inactivation of the Nab4 factor, which functions in poly(A) cleavage site selection, induces the formation of *SDH4* extended transcripts, strongly suggesting that Cth2Δ1-86 binding to *SDH4* mRNA in response to Fe deficiency promotes alternative 3′ end processing [58]. It is interesting to note that, similarly to Cth2, Nab4 is a shuttling protein that is retained in the transcript after 3′ end processing and export to the cytoplasm [56,59]. A more in-depth analysis by quantitative RT-PCR using primer pairs located up- and downstream of the normal poly(A) site showed that 3′ readthrough *SDH4* transcripts are also present in wild-type cells, be it less abundantly than in the Cth2Δ1-86 and *nab4* mutants [58]. By using yeast cells co-expressing wild-type Cth2 and truncated Cth2Δ1-86 proteins, Prouteau and coworkers demonstrated that the low abundance of extended *SDH4* transcripts in wild-type cells is due to its rapid degradation by a 5′ to 3′ mRNA decay mechanism [58], although other surveillance mechanisms in the nucleus can also contribute to its instability [60]. Other ARE-containing mRNAs regulated by Cth2, such as *ACO1* and mitochondrial cytochrome-c peroxidase *CCP1*, also exhibit 3′ readthrough enhanced by the deletion of Cth2 CR1 domain, suggesting that this may be a general strategy to post-transcriptionally down-regulate the expression of ARE-containing mRNAs when Fe availability is scarce [58]. These observations are consistent with a model in which Cth2 binding to the AREs that overlap with normal poly(A) sites would competitively inhibit the access of the 3′ end processing factors to the canonical sites for cleavage and polyadenylation, leading to the synthesis of alternative extended poly(A) transcripts (Figure 1). The Cth2 amino-terminal CR1 region can facilitate the recruitment of a 5′ to 3′ mRNA decay machinery (to be deciphered), which would trigger the rapid turnover of these extended transcripts. Interestingly, both the wild-type and Cth2Δ1-86 mutant cells, but not those cells lacking *CTH2*, accumulate high levels of *SDH2* extended transcripts, a Cth2-target encoding an Fe-S protein subunit of the succinate dehydrogenase [58]. These results suggest that Cth2 promotes the synthesis of *SDH2* extended transcripts, but neither promotes its degradation nor is independent of the Cth2 CR1 domain. Further studies are necessary to elucidate at which stage of the gene expression process and how Cth2 communicates with the 3′ end processing and decay machinery.

### 2.4. Cth2 Stimulates 5′ to 3′ Cytoplasmic Degradation of ARE-Containing mRNAs

Cytoplasmic degradation of eukaryotic mRNAs occurs by two general pathways, both of which initiate with the shortening of the 3′ poly(A) tail by the cytoplasmic Ccr4/Pop2/Not deadenylase complex. Following deadenylation, mRNAs can be subjected to 3′ to 5′ degradation by the cytoplasmic exosome (a conserved protein complex that exhibits 3′ to 5′ exonuclease activity, see below) or, more commonly in yeast, the 5′ cap can be removed by the Dcp1/Dcp2 decapping complex, followed by the 5′ to 3′ degradation of the transcript by cytoplasmic exonuclease Xrn1 (reviewed in [61–63]). Several protein factors, including the conserved DEAD box RNA helicase Dhh1, Pat1, Edc1-3 and the Lsm1-7 complex, stimulate the decapping rate *in vivo*. Some of these decapping factors, such as Dhh1 and Pat1, can act as inhibitors of translation at different steps and interact with the decapping and decay machinery by connecting mRNA translation and turnover.

The overexpression of either *CTH1* or *CTH2* under Fe-sufficient conditions can lead to growth impairment, whereas the deletion or mutagenesis of the TZF motif rescues cell growth in the *CTH1*/*CTH2*-overerexpressing cells [35,41]. These results suggest that Cth1/Cth2-mediated toxicity may be the result of the excessive degradation of essential transcripts. If this were the case, deletion of the genes required for Cth2 AMD would also rescue the growth defect of the *CTH2-*overexpressing cells. By following this genetic approach, the Dhh1 RNA helicase has been identified as a strong candidate to cooperate with Cth2 in the AMD mechanism that triggers adaptation to Fe deficiency [41]. Two other results have confirmed the Dhh1 function in Cth2 AMD: first, yeast two-hybrid assays have revealed an *in vivo* interaction between the carboxy-terminal domain of Dhh1 helicase and the Cth2 RNA-binding protein; second, mRNA half-life experiments have established that Dhh1 contributes to *SDH4* mRNA destabilization under low Fe conditions [41]. Dhh1 is an RNA helicase that performs ATPase activity, which both represses translation initiation by competing with the initiation factors and activates decay by recruiting the decapping and 5′–3′ decay machinery [64–66]. The Dhh1 requirement for Cth2 AMD suggests that mRNA turnover proceeds from 5′ to 3′. To test this hypothesis, an oligo(G) track, which folds in a strong secondary structure that blocks the exonucleolytic digestion of mRNAs, was inserted into the 3′ UTR of the *SDH4* transcript. The intermediate mRNA degradation species trapped were consistent with a model in which Cth2 recruits Dhh1 to ARE-containing transcripts to preferentially promote cytoplasmic 5′ to 3′ mRNA turnover [41,58] (Figure 1).

Dhh1 interacts with the Dcp1/Dcp2 decapping complex and the scaffold protein Edc3, and assists the transition of mRNAs from the active translation pool to specific cytosolic mRNP foci, known as processing bodies (or P-bodies). The mRNAs in P-bodies are translationally repressed and can either be stored (and eventually return to the translating pool) or undergo decapping and 5′ to 3′ degradation by exonuclease Xrn1 (reviewed in [67,68]). The current model for P-body assembly in *S. cerevisiae* proposes that different preformed complexes are recruited to an mRNA. The Dcp1/Dcp2/Dhh1/Edc3 complex binds to the 5′ cap and the Pat1/Lsm1-7/Xrn1 complex binds to the 3′ end. Interactions between members of these complexes lead to the formation of a closed-loop mRNP structure that assembles in larger aggregates to reach visible cytoplasmic granules. Thus, in response to certain stresses (glucose starvation, osmotic stress, stationary phase) or strains with defective mRNA decay machinery (*dcp1Δ*, *dcp2Δ*, *xrn1Δ*, *lsm1Δ*), the rate of transcripts being degraded or translationally inhibited increases up to a threshold that triggers the accumulation of P-bodies [64,69–71]. This is not the case of Fe deficiency in which P-bodies are not observed [41]. Nonetheless, it has been established that the aggregation of the mRNA decay components in P-bodies is not necessary for their function, but is rather a consequence [72]. However, it is important to highlight that the Cth2 protein is trapped in cytoplasmic P-bodies in the *dcp1Δ*, *dcp2Δ* and *xrn1Δ* yeast mutants, but not in an *lsm1Δ* strain [41]. These results corroborate the involvement of the Dcp1 and Dcp2 decapping enzymes and the Xrn1 exonuclease in Cth2 AMD, and suggest that the Cth2 protein cannot be released from decay machinery (P-bodies in this case) until the target mRNA has been degraded (Figure 1).

Cth2 TZFs alone are not sufficient to destabilize *SDH4* mRNA in response to Fe limitation, indicating that at least another Cth2 region is required for AMD [58]. As detailed above, the deletion of the first 86 conserved amino acids of the Cth2 protein (CR1 domain) partially impairs *SDH4* degradation, which strongly suggests that this region is important for Cth2 AMD [58]. Subcellular localization experiments with the Cth2Δ1-86 protein have shown that the truncation of Cth2 amino terminus does not affect protein shuttling [42]. Although further experiments are required, these results indicate that the Cth2Δ1-86 protein may bind ARE-containing mRNAs, but can display defects in the recruitment of the mRNA decay machinery to the Cth2-targeted transcript, leading to the accumulation of normal and extended *SDH4* transcripts. It would be interesting to ascertain whether the cells expressing the Cth2Δ1-86 protein exhibit a growth defect under low Fe conditions, as observed for those cells lacking *CTH2* or expressing a TZF-mutant allele.

### 2.5. Post-Transcriptional Regulation of CTH2 mRNA

As described above, the 3′ ends of the vast majority of *S. cerevisiae* mRNAs are generated by co-transcriptional cleavage and polyadenylation by the pre-mRNA processing machinery. However, there are a few examples of mRNAs (for instance, *NAB2* and *CTH2*) that escape this canonical 3′ end processing pathway [73–76]. When the Rna14, Rna15 or Pap1 components of the canonical 3′ maturation machinery are defective, most mRNAs are degraded by surveillance mechanisms. Yet in such circumstances, the synthesis of *CTH2* mRNA, which encodes the post-transcriptional regulator of Fe homeostasis, is not abolished, which suggests that the *CTH2* transcript is generated by a different 3′ end processing pathway [73]. Consistently with this hypothesis, the poly(A) sites of a mature *CTH2* mRNA locate to a noncanonical (GU_3–5_)_5_ repeat sequence at around 240 nucleotides downstream of the *CTH2* coding sequence in a region lacking the essential elements required at poly(A) sites such as the PE. It is important to mention that the (GU_3–5_)_5_ element does not function as a transcriptional terminator [73].

**Figure 2:**
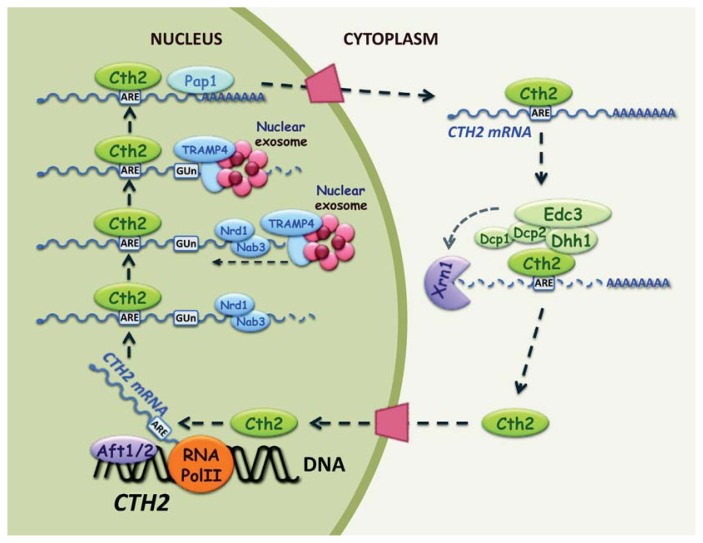
Model representing the post-transcriptional mechanisms regulating the *CTH2* mRNA expression. *CTH2* mRNA maturation starts with a 3′ extended transcript that is recognized by the Nrd1-Nab3 complex, and is subsequently processed by the nuclear exosome and the TRAMP4 polyadenylation complex. Eventually, the exosome pauses at a (GU_3–5_)_5_ site and Pap1 polyadenylates the trimmed *CTH2* transcript. Moreover, *CTH2* mRNA contains an ARE in its 3′ UTR which allows the binding of the Cth2 protein and auto-degradation. The auto-destabilization mechanism can be similar to other ARE-containing mRNAs and depends on the cytoplasmic 5′ to 3′ mRNA degradation machinery. Although not represented, Rat1 has also been implicated in the *CTH2* transcript turnover.

The exosome is an evolutionary conserved complex of exoribonucleases that is located in both the nucleus and the cytoplasm, and it participates in the 3′ end maturation and/or quality control of almost every RNA molecule in the cell (reviewed in [77–79]). The exosome core contains nine essential, yet catalytically inactive subunits. 3′ to 5′ exonuclease activity depends on the nuclear and cytoplasmic Dis3/Rrp44 exonuclease and on the exclusively nuclear Rrp6 exonuclease. The Nrd1-Nab3-Sen1 and the Trf4/5-Air1/2-Mtr4 (TRAMP) polyadenylation complexes assist the nuclear exosome core to define the targets that are to be processed and/or degraded. The Nrd1-Nab3-Sen1 complex interacts with the exosome to promote a proper 3′ end formation of several noncoding transcripts. Nrd1 and Nab3 act as RNA-binding sensors, which, together with Sen1 helicase, detect specific terminator sequences in the nascent transcripts that are targeted to the TRAMP complex. TRAMP is the major nuclear exosome cofactor. It can assemble into two distinct subcomplexes: TRAMP4, which includes the Trf4, Air1/2 and Mtr4 proteins, and TRAMP5 with Trf5, Air1/2, and Mtr4. Shortly after the Air1 and Air2 RNA-binding proteins recognize the RNA substrates, the Mtr4 RNA-dependent adenosine triphosphatase recruits the nuclear exosome, then Trf4 and Trf5 poly(A) polymerases add a short oligo(A) tail, which facilitates degradation by the exosome. Various RNA analyses have shown that the yeast cells defective in subunits of the core exosome or the nuclear exonuclease Rrp6 accumulate *CTH2* mRNAs, which extend 1.85 Kb beyond the *CTH2* translation termination codon, whereas this is not observed in those cells with a defective cytoplasmic exosome [73,80]. These results strongly suggest that the nuclear exosome participates in *CTH2* pre-mRNA processing. Moreover, the *air1Δair2Δ* and *trf4Δ* mutant cells, but not *trf5Δ*, accumulate extended *CTH2* mRNAs, indicating that the TRAMP4 complex may assist the exosome in the 3′ trimming of *CTH2* pre-mRNA. Finally, the yeast cells defective in the components of the Nrd1-Nab3-Sen1 nuclear exosome cofactor also accumulate 3′ extended *CTH2* mRNAs [73]. In line with this, the *CTH2* pre-mRNA sequence displays multiple consensus-binding sites for Nrd1 and Nab3 within its 3′ UTR. Furthermore, recent studies using an *in vivo* cross-linking approach to map binding sites for the components of the yeast non-poly(A) termination pathway have demonstrated that both Nrd1 and Nab3 bind *CTH2* extended pre-mRNAs [81,82]. All these data indicate that *CTH2* mRNA maturation does not follow the normal cleavage and polyadenylation process. Instead, they suggest that a 3′ extended *CTH2* transcript is recognized by the Nrd1-Nab3-Sen1 complex and is subsequently processed by the recruited TRAMP4 polyadenylation complex and the nuclear exosome (Figure 2). The mechanism that allows the exosome to pause at the (GU_3–5_)_5_ site is currently unknown. Analyses of *CTH2* poly(A) species have shown that Rna14 and Pap1 are required for the polyadenylation of the trimmed transcript [73]. It would be interesting to ascertain whether this novel mechanism of *CTH2* pre-mRNA processing also functions when *CTH2* transcription is dramatically induced by Fe deficiency.

In addition to the unusual 3′ end processing, *CTH2* mRNA contains a consensus ARE located 46 nucleotides downstream of its translation termination codon, which tightly modulates its abundance [73,83]. RNA expression analyses have shown that the deletion or mutagenesis of this ARE increases the *CTH2* transcript levels, especially under Fe-deficient conditions [73,83]. Yeast three-hybrid assays have demonstrated that both the Cth1 and Cth2 proteins specifically bind *in vivo* to *CTH2* transcript ARE [83]. Furthermore, the wild-type, but not the ARE-mutated 3′-UTR of *CTH2* mRNA, confers Cth2-dependent Fe regulation to *GCN4*, an Fe-independent regulated transcript [83]. These and other results demonstrate that the Cth2 protein specifically binds *in vivo* to an ARE located at the 3′ UTR of its own transcript in a negative-feedback auto-regulatory loop that limits its expression. Degradation of the *CTH2* transcript may proceed by the same cytosolic mechanism as other ARE-containing mRNAs, although the nuclear Rat1 exonuclease has also been implicated in this process [73] (Figure 2). The yeast cells lacking a functional *CTH2* ARE (CTH2-AREmt strain) display two phenotypes, which highlight the physiological relevance of this feedback regulatory mechanism. First under low Fe conditions, the Cth2-AREmt cells accumulate higher Cth2 mRNA and protein levels than the wild-type cells, which leads to a major growth defect [83]. As mentioned above, previous studies have already reported that the *CTH2* overexpression is toxic for yeast cells, although the underlying molecular mechanism is still unknown [35,41]. Second, many studies have shown that the simultaneous activation of both transcription and degradation permits more rapid, sensitive changes in gene expression when cells are confronted to environmental stresses (reviewed in [84]). This seems to be the case of *CTH2* since CTH2-AREmt cells display a considerable delay in resuming growth during the shift from Fe-deficient to Fe-sufficient conditions as compared to wild-type cells. Numerous data indicate that this delay is due to a defect in Cth2 mRNA and protein down-regulation in the early stages of Fe sufficiency, which limits the expression of Fe-dependent processes, including respiration, which are crucial for growth recovery [83]. Finally, *CTH1* mRNA also contains functional AREs within its 3′ UTR that modulate its abundance. Yeast three-hybrid and RNA analyses have shown that both the Cth1 and Cth2 proteins specifically bind *in vivo* to *CTH1* 3′ UTR to promote its transcript degradation [83]. This post-transcriptional regulation determines the expression pattern displayed by Cth1 during the progress of Fe deficiency. During the first hours of Fe deprivation, the *CTH1* expression is activated by transcription factors Aft1 and Aft2 and is limited by its own auto-degradation. When Fe limitation progresses and the Cth2 protein levels dramatically increase, *CTH1* mRNA abundance is reduced by Cth2 cross-regulation. Consequently, Cth1 expression is limited to the initial Fe deficiency stages when the transcripts involved in respiration and other mitochondrial processes are first targeted for degradation. If Fe limitation persists, Cth2 takes over by down-regulating the mRNAs implicated in other nonessential Fe-dependent processes and vacuolar Fe storage, while controlling its own expression at the same time.

### 2.6. Multilayered Regulation of Gene Expression by Mammalian TZF-Containing Proteins: Their Contribution to Fe Homeostasis

In addition to budding yeast, many eukaryotes ranging from invertebrates to plants and mammals possess similar TZF-containing proteins to Cth1 and Cth2. Mammals express at least three different TZF-family members, where tristetraprolin, also known as TTP, is the most extensively studied. The work done by many groups over the past two decades has established that TTP binds to the AREs present in the 3′ UTR of multiple mRNAs, including pro-inflammatory cytokine tumor necrosis factor α (*TNFα*), granulocyte-macrophage colony-stimulating factor (*GM-CSF*), cyclooxygenase 2 (*COX2*), several interleukins (*IL-2*, *IL-3*, *IL-6*), and proto-oncogenes (*c-Fos*, c-*Myc*), and that it promotes their turnover (reviewed in [85–87]). The TTP-mediated AMD mechanism involves the tethering of several components of the cytosolic mRNA decay machinery, including deadenylases, the decapping enzyme and the cytoplasmic exosome complex, onto the target transcript. TTP interacts first in an RNA-independent manner with the poly(A) binding protein PABP, which is responsible for protecting the poly(A) tail from degradation. This probably induces the displacement of PABP from the poly(A) tail and initiates the recruitment of the cytoplasmic Ccr4/Pop2/Not deadenylase complex to the transcript. Although no data are currently available, it is likely that mRNA deadenylation is also required for the 5′ to 3′ degradation of the Cth2 mRNA targets in yeast. TTP-mediated decay can occur through both the 5′ to 3′ and the 3′ to 5′ degradation pathways. TTP interacts and recruits decapping enzymes Dcp1/Dcp2 and cytoplasmic 5′ to 3′ exoribonuclease Xrn1 to the target transcripts by promoting their decapping and 5′ mRNA degradation. TTP can also interact with different components of the exosome by recruiting it to its target mRNAs to mediate the 3′ to 5′ turnover in the cytoplasm. The mechanism that uses TTP to target an mRNA toward one pathway or another is not known but, as with yeast Cth2, it seems that TTP predominantly uses the 5′ to 3′ decay pathway. Given its interaction with the 5′ to 3′ decay components (including Dcp1, Dcp2 and Xrn1), TTP can direct the localization of ARE-containing mRNA targets to P-bodies by acting as a nucleator of P-body formation by recruiting other RNPs. Additionally, TTP can localize to stress granules (SGs), which are cytoplasmic granules that form in response to different cellular stresses and which contain untranslated mRNAs stalled in the pre-initiation complex of ribosome assembly. TTP can shuttle between P-bodies and SGs, in accordance with its interaction with transportin, a member of the importin β-family, and 14-3-3 proteins, which exclude the TTP from the SGs preventing AMD. Therefore, TTP would determine the fate of ARE transcripts by escorting them to P-bodies to undergo decay or to stress granules in order to arrest their translation.

The implication of TTP in translational regulation is further supported by recent data [88–90]. Luciferase-based reporter and polyribosome fractionation experiments have shown that TTP specifically inhibits the translation of ARE-containing transcripts [90]. Moreover, TTP knockout and overexpression studies in macrophages have indicated that TTP functions as a repressor of TNFα mRNA translation [90]. RNA helicase RCK/p54/Dhh1, a general translational repressor (see above), directly interacts with TTP to regulate ARE-mRNA translation [90]. As described above, the yeast Dhh1 interaction with Cth2 is essential for Cth2-mediated AMD. However, a possible role for Cth2 in translational regulation remains to be addressed. The translational fate of TNFα mRNA depends on the presence of the RNA-binding protein HuR, which competes with TTP for binding to the ARE in a process regulated by the p38 MAPK (mitogen-activated protein kinase) pathway [88]. Its downstream target, the MK2 kinase, phosphorylates both TTP and HuR by facilitating the replacement of TTP with HuR at the ARE. Whereas TTP phosphorylation diminishes its binding to the ARE, HuR phosphorylation facilitates its translocation to the cytoplasm, where it promotes the initiation of translation by enhancing the association of TNFα with the endoplasmic reticulum polysome pool [88]. Many aspects of the TTP translational regulation mechanism remain to be elucidated, such as when and how TTP interacts with the translation machinery. It has been speculated that the interaction between TTP and PABP might interfere with the PABP-eIF4G-eIF4E-mediated mRNA circularization, which is required for translation, whereas the phosphorylation of PABP, also an MK2 substrate, may weaken the PABP-TTP interaction and allow its replacement with HuR [88]. Recent results have shown that the recruitment of Cul4B, a scaffolding component of the Cullin ring finger ligase family of ubiquitin E3 ligases, to the TTP-containing mRNP promotes translation [89], indicating that the TTP function as a translational regulator is more complex than initially anticipated, and that further characterization is necessary.

Similarly to yeast Cth2, mammalian TTP shuttles between the nucleus and the cytoplasm [91,92]. An NLS located between the two zinc fingers facilitates nuclear import, whereas amino-terminal NES triggers its Crm1-dependent export to the cytoplasm. The relative subcellular distribution of TTP differs according to cell type and extracellular signals. A recent study has revealed a novel nuclear function for mammalian Tis11b, a TZF-containing protein that mainly localizes to the nuclei of endothelial cells [93]. As previously shown for yeast Cth2, Tis11b regulates gene expression by interfering with normal 3′ end processing. Tis11b preferentially binds to an ARE located at the poly(A) site of an angiogenic mRNA, called *DII4*, by interfering with normal mRNA cleavage and transcript efficiency, and by producing 3′ readthrough transcripts [93]. Although the mechanism by which Tis11b down-regulates the *DII4* expression has not yet been deciphered, the extended transcripts resulting from defective polyadenylation are probably degraded by the nuclear surveillance pathway through the 3′ exonucleolytic activity of the nuclear exosome.

Multiple signaling pathways and environmental cues regulate the TTP expression and function. As recently described for Cth2, mammalian TTP can also bind AREs within its own 3′ UTR, thus adding an auto-regulatory negative feedback loop that could eventually regulate both its decay and its translation [88,94,95]. Interestingly, the mRNAs encoding the two other TZF-proteins expressed in humans also contain AREs, which hints at the possibility of a cross-regulation between different TZF members, as previously shown for yeast Cth1 and Cth2 [83]. Among several pathways, p38 MAPK is critical for TTP regulation. MK2 inhibits the TTP function in AMD by phosphorylating two specific serine residues, which serve as docking sites for multifunctional adaptor 14-3-3 proteins. Upon the 14-3-3 binding to the phosphorylated serines, the stability and cytoplasmic localization of the TTP protein increases and blocks its dephosphorylation by the PP2A protein phosphatase (reviewed in [13]). More recent results have shown that TTP phosphorylation also prevents the recruitment of mRNA deadenylases to the target transcript in a 14-3-3-independent manner [96,97]. However, our current understanding of the role of TTP phosphorylation in this function is incomplete because phosphorylated TTP still functions in AMD in some physiological circumstances [98]. Interestingly, the yeast Cth2 protein exhibits doublet bands in Western blot analyses due to phosphorylation [42]. The specific Cth2 phosphorylation sites are probably located in the conserved CR1 region as the Cth2Δ1-86 proteins have lost the shifted band. Furthermore, Cth2 phosphorylation seems sensitive to Cth2 subcellular localization, and is hypophosphorylated when forced to enter the nucleus and hyperphosphorylated once inside the cytoplasm. Elucidating the role played by phosphorylation in Cth2 can contribute to our understanding of the post-translational mechanisms regulating TZF-containing proteins.

Importantly**,** recent experiments in different mammalian cell lines and TTP knockout mice indicate that TTP plays an active role in the maintenance of mammalian cellular Fe homeostasis [99]. First, the expression of TTP and of other TZF-containing proteins in mouse embryonic fibroblasts (MEFs) is strongly induced by the addition of different Fe chelators. Second, TTP interacts *in vivo* with the 3′ UTR of TfR1 mRNA by promoting its destabilization. Thus, the TfR1 protein levels in TTP knockout mice are higher than in wild-type animals, leading to Fe accumulation. Furthermore, TTP reduces the expression of other Fe proteins that contain AREs in their 3′ UTR transcripts, such as ABCE1 (an Fe-S cluster protein involved in ribosome biogenesis) and Lias (lipoic acid synthase), whose budding yeast homologs are regulated by Cth2. Third, as compared to wild-type cells, TTP knockout MEF cells display severely reduced cell viability when Fe availability diminishes, which underlines the biological relevance of this regulation. Fourth and finally, the expression of a wild-type, but not a TZF-mutant TTP protein, in *cth1Δcth2Δ* yeast cells promotes the down-regulation of *SDH4* and *ACO1* mRNAs, suggesting a functional conservation between budding yeast and mammals. Taken together, these observations are consistent with a model in which TTP functions in optimizing cellular Fe utilization when its availability diminishes. Importantly, the cellular energy sensor mTOR, which is active under energetically favorable conditions and becomes inhibited under stressful conditions, including nutrient starvation and DNA damage, also modulates cellular Fe homeostasis by directly inhibiting the TTP function [99]. Interestingly, treatment of yeast cells with rapamycin, a specific TOR inhibitor, induces the expression of *CTH1* and *CTH2* mRNAs and opens up the possibility of the nutritional status of the cell also influencing Fe utilization in *S. cerevisiae*.

## 3. Post-Transcriptional Regulation in Response to Iron Excess

In addition to the activation of multiple antioxidant defense systems to combat oxidative stress, yeast cells have developed sophisticated strategies that detoxify cytosolic Fe and limit Fe acquisition when concentrations are too high. In response to Fe excess, transcription factors Aft1 and Aft2 are released from the FeREs and are translocated to the cytoplasm, thus being unable to activate the expression of the Fe acquisition and mobilization systems [19–21]. When the Fe concentration rises, Fe uptake is also inhibited by ubiquitination, endocytosis and degradation of Ftr1 protein, an essential component of the high affinity Fe uptake complex in the plasma membrane [100]. Moreover, the Yap5 transcription factor activates the expression of the Ccc1 vacuolar importer to allow Fe storage [27,28]. A post-transcriptional regulatory mechanism mediated by the Rnt1 endoribonuclease also protects budding yeast cells from Fe toxicity by limiting the expression of a subset of genes from the Fe acquisition system [101].

Rnt1 is a class I RNase III nuclear endoribonuclease constituted by an RNase III catalytic domain and a double-stranded RNA-binding domain (dsRBD). The conformational flexibility of Rnt1 dsRBD allows the specific recognition of the double-stranded RNA hairpin tetraloop structures capped by a conserved (A/u)GNN motif, whereas its RNase III domain triggers mRNA cleavage to generate 5′-monophosphate and 3′ hydroxyl termini with a two-base 3′ overhang [102–107]. Rnt1 cleavage products are usually further processed in the nucleus by the Rat1 5′ exonuclease and the 3′ exosome. However, a recent study has indicated that nuclear degradation is not the default fate of Rnt1-cleaved RNAs [108]. By inserting an Rnt1 target site into a reporter transcript, these authors have observed that the cleavage products are exported and degraded in the cytoplasm by the Xrn1 5′ exoribonuclease and the cytosolic exosome, suggesting that specific elements within the transcript contribute to determine the final destination of the cleavage products. Rnt1 functions in a large number of mRNA processing and degradation pathways, including maturation of rRNA and a variety of small RNAs, such as snRNAs and snoRNAs, and mRNA quality control through cleavage within introns of unspliced pre-mRNAs. Interestingly, recent reports have demonstrated that Rnt1 is also involved in polyadenylation-independent transcription termination by cleaving many transcripts downstream of the canonical poly(A) site, thus preventing the transcription read-through of inefficient 3′ processing ends [109,110]. Rnt1 also post-transcriptionally regulates the expression of genes in various cellular pathways by directly cleaving the stem-loop structure sites located in the coding sequence of target mRNAs. For instance, Rnt1 binds and promotes the degradation of the mRNAs encoding the glucose-dependent repressor Mig2, aci-reductone dioxygenase Adi1, involved in the methionine salvage pathway, multiple subunits of telomerase, and proteins associated with the morphogenesis checkpoint and the cell wall integrity pathway [111–114]. Consistently with this negative regulation, *rnt1Δ* cells exhibit a delay in the G1 phase of the cell cycle, sensitivity to stresses related to the cell wall integrity pathway, and elongated telomeric repeat tracks [113–115].

**Figure 3:**
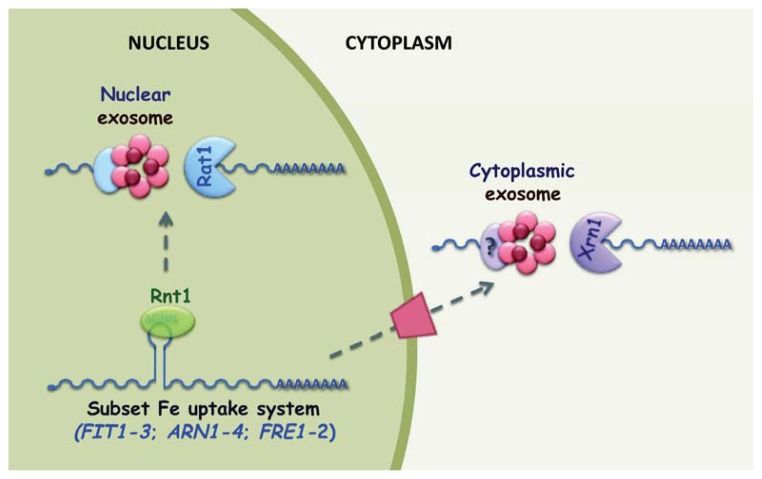
Upon Fe excess, *S. cerevisiae* RNase III endonuclease Rnt1 limits the expression of a subset of genes from the Fe uptake system to prevent Fe toxicity. Rnt1 specifically binds to the stem-loop structures present in some mRNAs from the Fe regulon. The nuclear exosome, and the Rat1 and Xrn1 5′ to 3′ exonucleases, rapidly degrade Rnt1 cleavage products. Despite not being demonstrated, the cytoplasmic exosome has also been included in this model.

Genome-wide expression analyses conducted under Fe-replete conditions have revealed that the expression of a subset of genes from the Fe regulon is up-regulated in budding cells lacking Rnt1 endoribonuclease [101]. These mRNAs encode the proteins implicated in Fe siderophore uptake, such as cell wall mannoproteins Fit1-3 and siderophore transporters Arn1-4, the metalloreductases Fre2 and Fre3, the proteins that participate in the Fe-S cluster assembly pathway, such as Isu1 and Isu2, and the RNA-binding protein Cth2. The genes encoding the Fet3 and Ftr1 high-affinity reductive uptake system and the Fre1 metalloreductase are not up-regulated in the Fe-replete *rnt1Δ* cells. Bioinformatics analyses have predicted that stem-loop structures capped with (A/u)GNN-type tetraloops can potentially assemble within the coding sequence of various Fe uptake genes whose expression is altered in *rnt1Δ* cells. In line with this prediction, *in vitro* experiments done with purified recombinant Rnt1 protein have demonstrated that the stem-loop structures within the *FIT2* and *ARN2* genes are directly recognized and cleaved by wild-type Rnt1, but not by the catalytically inactive Rnt1-E320K mutant form [101]. Furthermore, deletion of the Rnt1 canonical recognition sequence in *FIT1* mRNA slightly increases its abundance in Fe-sufficient cells [101]. Taken together, these results strongly suggest that the Rnt1 regulation of this subset of genes from the Fe starvation response is a direct effect rather than a secondary Fe-sensing effect. In the absence of Rnt1 endoribonuclease, multiple Fe uptake genes, such as *FIT1*, *FIT2*, *ARN1*, *ARN2*, *FRE2* and *FRE3*, accumulate extended transcript species. Other studies with various mRNA degradation mutants have shown that the nuclear exosome, and nuclear Rat1 and cytosolic Xrn1 5′ to 3′ exoribonucleases, cooperate to rapidly degrade Rnt1 cleavage products [101] (Figure 3). Importantly, the yeast cells lacking *RNT1* are hypersensitive to Fe excess since they are unable to grow in media with high concentrations of this metal [101]. Therefore, Rnt1 endoribonuclease plays a protective role when extracellular Fe exceeds cellular needs by promoting the degradation of members of the Fe acquisition machinery. Furthermore, accumulation of *CTH2* mRNA in *rnt1Δ* cells under Fe-sufficient conditions can also be detrimental because it could inhibit the recovery of crucial Fe-dependent processes, including respiration. As detailed above, budding yeast cells possess auto-regulatory mechanisms to rapidly down-regulate the *CTH2* mRNA levels when Fe availability increases in order to allow the Fe-requiring pathways to resume [83]. The Rnt1-mediated degradation of *CTH2* mRNA under Fe-sufficient conditions can also contribute to the recovery of Fe-dependent metabolism.

Interestingly, Rnt1 is also important for the activation of the Aft1/2-dependent Fe regulon that occurs when Fe availability decreases. The cells lacking *RNT1* or expressing the catalytically inactive Rnt1-E320K mutant exhibit a considerable delay in the activation of many members of the Fe regulon, including *FET3*, *FTR1*, *FRE1-3*, *FIT1-3* and *ARN2-3* [101]. The nuclear localization of Rnt1 is required for this function since an Rnt1-truncated version lacking its NLS fails to activate the Fe starvation response [101]. Although the underlying mechanism is currently unknown, different hypotheses have been proposed to explain the defect that *rnt1Δ* cells display in activating the Fe regulon. One possible explanation is that the increase in the Fe uptake system observed in *rnt1Δ* cells leads to higher endogenous Fe levels. Thus, when Fe availability diminishes, *rnt1Δ* mutants could take longer than wild-type cells to deplete intracellular Fe stores and, therefore, the Fe starvation response would be delayed. Another explanation could be that the up-regulation of *ISU1* and *ISU2*, which encode proteins involved in the Fe-S cluster synthesis pathway, observed in *rnt1Δ* mutants increases the Fe-S formation rate to then inhibit Aft1 translocation to the nucleus. The drop in *FET3* transcription observed in the *rnt1Δ* cells expressing a FET3-LacZ construct is consistent with both hypotheses. However, the determination of the endogenous Fe levels could help distinguish them. It would be interesting to ascertain whether the defect in the Fe regulon activation observed in *rnt1Δ* cells leads to increased sensitivity to Fe deficiency.

## 4. Conclusions

Post-transcriptional regulation has emerged as an important mechanism for the rapid and flexible control of gene expression, especially in response to environmental stresses. The relevance of post-transcriptional mechanisms in Fe homeostasis control is evidenced by the large number of regulatory systems conserved in different organisms under both low and high Fe conditions. The Cth2 protein performs an essential function in budding yeast metabolic adaptation to Fe deficiency by targeting many ARE-containing transcripts for degradation. The mechanisms that Cth2 uses to promote mRNA turnover are diverse and include alternative 3′ end processing and cytoplasmic AMD. Most studies have focused on the degradation of a few Cth2 targets, mainly *SDH4* mRNA. However, there is some evidence for other mechanisms of the Cth2-mediated mRNA regulation function in *S. cerevisiae* [41,58,73]. Another important feature of Cth2-mediated regulation is the tight control of its expression levels. Budding yeast cells simultaneously regulate both the transcription and degradation of the *CTH2* transcript by modulating the function of the Aft1/Aft2 and Cth1/Cth2 regulatory factors, respectively. The physiological significance of this fine-tuned regulation is highlighted by the growth defects displayed by the cells lacking either *CTH2* transcription activation or mRNA degradation. As discussed in this review, many questions about the Cth2 mechanism of action still remain to be answered. The further characterization of the Cth2 function in budding yeast cells will contribute to our understanding of the mechanisms that eukaryotic TZF-containing proteins utilize to post-translationally regulate gene expression.
